# First Principles Study of the Structure–Performance Relation of Pristine W_n+1_C_n_ and Oxygen-Functionalized W_n+1_C_n_O_2_ MXenes as Cathode Catalysts for Li-O_2_ Batteries

**DOI:** 10.3390/nano14080666

**Published:** 2024-04-11

**Authors:** Liwei Zhu, Jiajun Wang, Jie Liu, Ruxin Wang, Meixin Lin, Tao Wang, Yuchao Zhen, Jing Xu, Lianming Zhao

**Affiliations:** School of Materials Science and Engineering, China University of Petroleum (East China), Qingdao 266580, China; 2114010430@s.upc.edu.cn (L.Z.); lw13255323168@163.com (J.W.); 2114011202@s.upc.edu.cn (J.L.); 2214010605@s.upc.edu.cn (R.W.); linmeixin5683@163.com (M.L.); wangtaoqwas@163.com (T.W.); 15688469531@163.com (Y.Z.)

**Keywords:** density functional theory, electrocatalysis, oxygen reduction reaction, oxygen evolution reaction

## Abstract

Li-O_2_ batteries are considered a highly promising energy storage solution. However, their practical implementation is hindered by the sluggish kinetics of the oxygen reduction (ORR) and oxygen evolution (OER) reactions at cathodes during discharging and charging, respectively. In this work, we investigated the catalytic performance of W_n+1_C_n_ and W_n+1_C_n_O_2_ MXenes (n = 1, 2, and 3) as cathodes for Li-O_2_ batteries using first principles calculations. Both W_n+1_C_n_ and W_n+1_C_n_O_2_ MXenes show high conductivity, and their conductivity is further enhanced with increasing atomic layers, as reflected by the elevated density of states at the Fermi level. The oxygen functionalization can change the electronic properties of WC MXenes from the electrophilic W surface of W_n+1_C_n_ to the nucleophilic O surface of W_n+1_C_n_O_2_, which is beneficial for the activation of the Li-O bond, and thus promotes the Li^+^ deintercalation during the charge–discharge process. On both W_n+1_C_n_ and W_n+1_C_n_O_2_, the rate-determining step (RDS) of ORR is the formation of the (Li_2_O)_2_* product, while the RDS of OER is the LiO_2_* decomposition. The overpotentials of ORR and OER are positively linearly correlated with the adsorption energy of the RDS Li_x_O_2_* intermediates. By lowering the energy band center, the oxygen functionalization and increasing atomic layers can effectively reduce the adsorption strength of the Li_x_O_2_* intermediates, thereby reducing the ORR and OER overpotentials. The W_4_C_3_O_2_ MXene shows immense potential as a cathode catalyst for Li-O_2_ batteries due to its outstanding conductivity and super-low ORR, OER, and total overpotentials (0.25, 0.38, and 0.63 V).

## 1. Introduction

Energy conversion and storage systems have become a critical component of the future energy sector as global energy demand continues to grow and energy transformation accelerates. Among these systems, Li-O_2_ batteries are considered one of the most promising solutions for energy storage due to their high energy density (up to 3500 Wh·kg^−1^), long lifespan, and environmental friendliness [1]. Li-O_2_ batteries typically are composed of lithium metal anodes, oxygen cathodes, and non-aqueous Li^+^ conductive electrolytes [2]. During discharge, atmospheric oxygen reacts with (Li^+^ + e^-−^) pairs to form (Li_2_O)_2_, which undergoes an oxygen reduction reaction (ORR) on the cathode:

(1) $$4{Li}^{+}+4e^{-}+O_{2}↔{(Li}_{2}{O)}_{2}$$

Upon charging, (Li_2_O)_2_ is converted back to (Li^+^ + e^−^) and O_2_. Therefore, the oxygen evolution reaction (OER) takes place at the cathode [3]. However, the sluggish kinetics of ORR and OER at the cathode lead to increased charge and discharge overpotentials, limited discharge capacity, and inadequate cycling performance of Li-O_2_ batteries, thereby limiting their practical applicability [4]. Furthermore, due to the poor conductivity of the discharge product Li_4_O_2_, its decomposition during charging requires a significant overpotential, resulting in the decomposition of the solvent at high voltage. Additionally, other components in the air, such as CO_2_ and H_2_O, may react with Li_2_O_2_ to form by-products, such as Li_2_CO_3_ and LiOH, which are more difficult to decompose. Finally, the battery reaches a fault state [5,6]. Consequently, the development and synthesis of efficient cathode catalysts play a pivotal role in enhancing the performance of Li-O_2_ batteries. To date, several categories of catalyst materials have been employed in Li-O_2_ batteries, including noble metals and their alloys (Pt [7] and Pt-Au alloys [8]), functional carbon materials (graphene [9] and carbon nanotube [10]), transition metal oxides (Mn_3_O_4_ [11] and Co_3_O_4_ [12]), transition metal carbides/nitrides (Mo_2_C [13] and MoN [1]), metal-organic frameworks (Ru-MOF-C [14] and Tz-Mg-MOF-74 [15]), and other composite structure electrocatalysts [16,17].

In recent years, MXenes have attracted considerable attention in the field of electrocatalysis due to their unique properties as two-dimensional (2D) layered nitride or carbide materials, including low resistivity, fast ion transport, and tunable interlayer structure [18]. MXenes are typically fabricated utilizing three layers of the MAX phase as a precursor, followed by etching the A layer via various techniques and modifying functional groups on the surface. The general formula for MAX phases is M_n+1_AX_n_. The synthesized MXenes can be represented as M_n+1_X_n_T_x_, where M is a transition metal (such as Cr, Ti, Mn, Mo, or W), A is usually a group 13 or 14 elements (such as Al, Si, Ga, or Ge), X is C or N, and T represents the end of surface (such as O, F, or OH). Nowadays, various MXenes have been used in Li-O_2_ batteries [19] and supercapacitors [20]. For example, Xu et al. reported that the O-terminated V_2_C MXene (V_2_CO_2_) can significantly reduce battery overpotential to 0.75 V, increase capacity to 8577 mAh g^−1^ at 100 mA g^−1^, and improve durability up to 302 cycles for Li-O_2_ batteries. The electrocatalytic activity of V_2_CO_2_ is enhanced by improving the affinity for the substrate with Li_2_O_2_ through O-termination. Moreover, the unique 2D V_2_CO_2_ structure exhibits remarkable conductivity and excellent mass transfer performance during battery operation, thereby effectively optimizing the dynamics of Li-O_2_ batteries [18]. The Nb_2_C MXene nano-sheets with uniform O-terminal surfaces were fabricated as a high-rate cathode for Li-O_2_ batteries by Li et al. [21]. This catalyst exhibits a large capacity of 19785.5 mAh g^−1^ and a high-rate stability of 130 cycles at 200 mA g^−1^ and 3 A g^−1^ [21]. Density functional theory (DFT) calculations indicate that the O-terminated Nb_2_C MXene can enhances its affinity with LiO_2_ and Li_2_O_2_, facilitating spatial orientation accumulation and stable decomposition of discharge products [21]. These findings highlight the significant potential of MXenes in Li-O_2_ batteries. However, further research is needed to elucidate the relationship between MXene structure and activity in Li-O_2_ batteries, especially regarding the influence of surface functional groups and the number of atomic layers of M_n+1_X_n_T_x_ MXene on catalytic performance.

In light of these formidable challenges, we systematically investigate the catalytic performance of WC-MXenes (i.e., W_n+1_C_n_ and W_n+1_C_n_O_2_, n = 1, 2, and 3) as cathode materials for Li-O_2_ batteries through first principles calculations. The WC-MXenes were selected for the following benefits: (a) W-based materials exhibit excellent mechanical strength, outstanding chemical stability, and high tolerance to acidic environments, and are naturally abundant and environmentally friendly [22,23]. (b) The W_x_C_y_ materials have been proven Pt-like catalytic features in many electrochemical reactions, such as ORR and OER [24]. (c) WC MXenes have a metallic nature, high charge capacity, and low Li^+^ diffusion barrier, facilitating the rapid deintercalation of Li^+^ during the charge–discharge process [25,26,27].

Herein, the electrochemical catalytic models of the WC MXene cathode were established to simulate OER and ORR during the charge–discharge process. The relationship between structure and catalytic performance of WC MXenes was revealed. In particular, the effects of surface oxygen functionalization and atomic layer number on the electronic structure of WC MXenes were explored to modulate the ORR and OER activities. We focused solely on the intrinsic properties of the electrocatalysts. External factors such as discharge product morphology, electrolyte decomposition, side reactions with CO_2_ and H_2_O, lithium metal corrosion, and oxygen electrode polarization were not considered in this work. Initially, the geometric and electronic structures of pristine and functionalized WC MXenes were investigated, revealing that both pristine W_n+1_C_n_ and O-functionalized W_n+1_C_n_O_2_ (n = 1, 2, and 3) show excellent electrical conductivity. Furthermore, the influence of atomic layer number and oxygen functional groups on the electronic properties and catalytic activity of WC MXenes was examined. Then, the formation and reversible decomposition of Li_x_O_2_ (x = 1, 2, and 4) were simulated during charge–discharge processes. Finally, the overpotentials of WC MXenes were quantitatively calculated to evaluate their catalytic performance for Li-O_2_ batteries. Our study demonstrates that oxygen functionalization can convert the electrophilic W surface of W_n+1_C_n_ to the nucleophilic O surface of W_n+1_C_n_O_2_, promoting the deintercalation of Li^+^ during the charge–discharge process. The ORR and OER overpotentials are positively linearly correlated with the adsorption energy of the Li_x_O_2_* intermediates. Surface oxygen functionalization and increasing atomic layers can weaken the Li_x_O_2_ adsorption by lowering the energy band center of WC MXenes, thereby reducing the ORR/OER overpotentials. Notably, the W_4_C_3_O_2_ MXene shows the superior catalytic performance, characterized by high conductivity and ultra-low ORR, OER, and total overpotentials (0.25, 0.38, and 0.63 V). This study bridges the gap left by WC MXenes as cathode catalysts for Li-O_2_ batteries and enriches the research of the MXene family in Li-O_2_ batteries.

## 2. Details of the Calculation

In this study, all spin-unrestricted DFT calculations were performed using the DMol^3^ module [28,29]. To describe electron exchange correlation, we employed the Perdew–Burke–Ernzerhof (PBE) functional based on the generalized gradient approximation (GGA) [30], which has been widely used in research on WC Mxenes [31,32]. However, it should be noted that GGA function fails to accurately describe the long-range van der Waals (vdW) interaction. Therefore, the Grimme’s dispersion correction (DFT-D3) method was incorporated into our study to account for vdW interactions [4]. The Grimme’s correction method consistently describes all chemically relevant elements within a periodic system and exhibits equal efficacy for both molecules and solids. Furthermore, it achieves a CCSD (T) accuracy, with an error of within 10% [33]. Therefore, the Grimme’s method provides a reliable description of the surface chemistry of MXene systems including the WC family [27,34].

The classical Monkhorst–Pack scheme is employed to generate K-points [35]. In the convergence test, a 4 × 4 × 1 Monkhorst–Pack grid was utilized for K-point sampling [36], and the self-consistent convergence criterion (SCF tolerance) was set to 1 × 10^−5^ Ha. To eliminate interlayer interactions, a vacuum layer with a thickness of 20 Å was introduced along the z direction of the WC MXene cell. During geometric optimization, equilibrium geometry was achieved when energy, force, and displacement fall below the thresholds of 2 × 10^−5^ Ha, 4 × 10^−3^ Ha Å^−1^, and 5 × 10^−3^ Å, respectively [37]. The smearing value of 0.005 Ha expedites the convergence rate of electronic structure optimization.

The formation energy ($E_{f}$) of W_n+1_C_n_ MXenes was defined as:

$$E_{f}=\left[E_{W_{n+1}C_{n}}-\left(n+1\right)E_{W}-nE_{C}\right]/(2n+1)$$

where $E_{W_{n+1}C_{n}}$ is the total energy of W_n+1_C_n_, $E_{W}$ is the chemical potential of one W atom in the bulk W, and $E_{C}$ is the chemical potential of one C atom in graphene.

The adsorption energy ($E_{ads}$) was defined as:

$$E_{ads}=E_{total}-E_{substrate}-E_{adsorbate}$$

where $E_{total}$ represents the total energy of the adsorption system, $E_{substrate}$ is the total energy of the substrate, and $E_{adsorbate}$ is the total energy of the adsorbate.

During the charge and discharge processes, the free energy change (Δ*G*) of the intermediates at each step can be described as follows:

$$\Delta G=E-E_{0}-\Delta n_{Li}\left(\mu_{Li}-eU\right)+\Delta n_{O_{2}}\mu_{O_{2}}$$

where E represents the total energy of the adsorption system at a specific reaction step, $E_{0}$ is the total energy of the adsorption system at the initial reaction step, and the $∆n_{Li}$ and $∆n_{O_{2}}$ are the numbers of Li^+^ and O_2_, respectively. The chemical potential $\left(\mu_{Li}\right)$ is defined as the energy of a Li atom in the bulk phase, and the chemical potential ($\mu_{O_{2}})$ is defined as the energy of an isolated O_2_ molecule in the gas phase. It has been observed that there is a computational error when calculating the binding energy of O_2_ molecules by using the DFT algorithm [38]. In this study, we determine the total energy of an O_2_ molecule in gas phase by combining experimental O_2_-binding energy (5.12 eV [39]) with DFT-calculated O atom energy. This is a widely adopted approach in the previous literature [40]. Moreover, any over-binding errors for oxygen molecules are expected to be compensated for free energy profiles of ORR and OER. Therefore, we can accurately determine the qualitative characteristics of free energy profiles of ORR and OER. The term *-eU* was included to describe changes in electron potential at potential *U*. Additionally, since formation and decomposition of Li_x_O_2_ occur under low temperatures (T) and pressures (P), effects such as entropy (-TS) and volume (PV) are disregarded, which is frequently used in studies in Li-O_2_ batteries [41,42]. In this work, we focused on examining the thermodynamic process of the elementary steps of ORR and OER. We assumed that any barriers between these steps are sufficiently small to not impose additional dynamic constraints on starting current at a measurable level. This approach has been widely employed in investigating Li-O_2_ batteries [43,44].

The ORR ($\eta_{ORR}$), OER ($\eta_{OER}$), and total ($\eta_{TOT}$) potentials are defined as $\eta_{ORR}=U_{0}-U_{DC}$, $\eta_{OER}=U_{C}-U_{0}$, and $\eta_{TOT}=\eta_{ORR}+\eta_{OER}$, respectively. In this definition, $U_{DC}$ represents the highest discharge potential that drives the energy downhill for all ORR steps, $U_{C}$ represents the lowest charge potential that drives the energy downhill for all OER steps, and $U_{0}$ denotes the equilibrium potential (∆G≤0) that facilitates the spontaneous occurrence of ORR/OER [45,46].

The d-band center ($\varepsilon_{d}$) and p-band center ($\varepsilon_{p}$) are calculated using formulas:

$$\varepsilon_{d}=\frac{\int_{-\infty }^{\infty }E\rho_{d}\left(E\right)dE}{\int_{-\infty }^{\infty }\rho_{d}\left(E\right)dE}$$

and

$$\varepsilon_{p}=\frac{\int_{-\infty }^{\infty }E\rho_{p}\left(E\right)dE}{\int_{-\infty }^{\infty }\rho_{p}\left(E\right)dE}$$

where $\rho_{d}\left(E\right)$ and $\rho_{p}\left(E\right)$ denote the densities of d-states and p-states at the energy level *E*, respectively.

## 3. Results and Discussion

### 3.1. Structural Properties of WC MXenes

**Figure 1:**
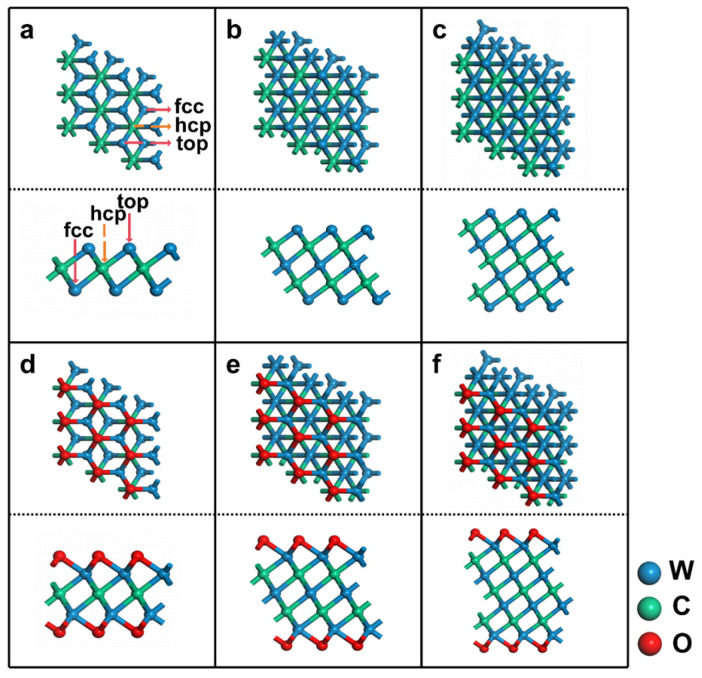
Top and side views of (**a**) W_2_C, (**b**) W_3_C_2_, (**c**) W_4_C_3_, (**d**) W_2_CO_2_, (**e**) W_3_C_2_O_2_, and (**f**) W_4_C_3_O_2_. Top, hcp, and fcc indicate possible adsorption sites.

The crystal structures of optimized W_2_C, W_3_C_2_ and W_4_C_3_ are depicted in Figure 1. W_2_C MXene exhibits a hexagonal structure similar to hexagonal MoS_2_ with two surface W layers and an intermediate C layer (Figure 1a). By altering the stacking sequence of the W and C layers according to ABA stacking, the W-C-W sandwich structure of W_2_C MXene serves as a foundation for constructing thicker MXenes such as W_3_C_2_ and W_4_C_3_ (Figure 1). Consequently, W_3_C_2_ MXene consists of three W layers and two C layers, while W_4_C_3_ MXene contains four W layers and three C layers. The lattice constants of W_2_C MXenes are calculated to be a = b = 2.83 Å, and the W-C bond length is found to be 2.126 Å, which is in excellent agreement with the previously reported results (Lattice constant a = b = 2.84 Å, and W-C bond length 2.130 Å) [27,34]. In addition, the formation energy of W_2_C, W_3_C_2_, and W_4_C_3_ MXenes is calculated to be −3.48, −3.35, and −3.33 eV (see Table S1), respectively, indicating their strong thermodynamic stability [25]. All of these indicate the reliability of the WC-MXene models and calculation methods.

The surface modification is commonly employed to introduce functional groups, such as -O, -F, and -OH, onto the MXene surfaces to improve the interaction between MXenes and adsorbents [47]. Previous studies have demonstrated that oxygen from air can replace -F and -OH groups, leading to more stable O-terminated MXenes. This suggests that WC MXenes are highly susceptible to being covered by -O groups [48,49,50]. Therefore, we selected W_2_C, W_3_C_2_, and W_4_C_3_ MXenes with full coverage of -O groups (i.e., W_2_CO_2_, W_3_C_2_O_2_ and W_4_C_3_O_2_, see Figure 1d–f) as probes for surface-functionalized WC MXenes.

**Figure 2:**
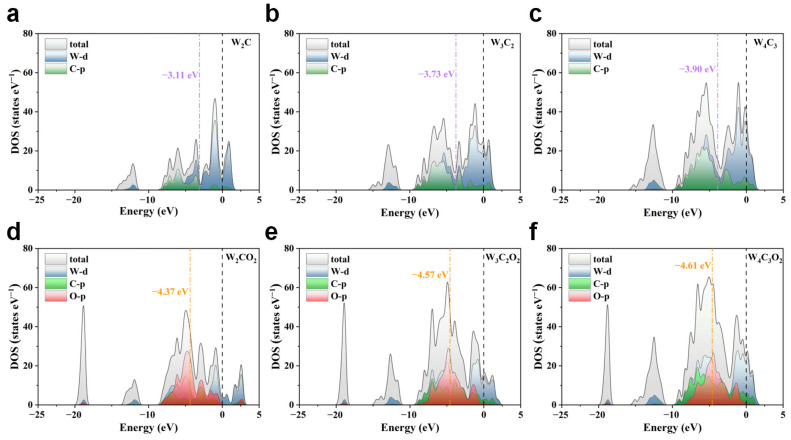
Projected density of states (PDOS) of (**a**–**c**) W_n+1_C_n_ and (**d**–**f**) W_n+1_C_n_O_2_. The Fermi level marked by the black dashed line is set as energy zero.

The projected state density (PDOS) of the pristine W_n+1_C_n_ and O-terminated W_n+1_C_n_O_2_ (n = 1, 2, 3) MXenes were plotted to investigate the electronic properties of WC MXenes, as depicted in Figure 2. It can be observed that the total density of states (TDOSs) of both W_n+1_C_n_ and W_n+1_C_n_O_2_ intersect with the Fermi level, indicating the high electrical conductivity of W_n+1_C_n_ and W_n+1_C_n_O_2_. With the increasing number of atomic layers of WC MXenes, the intensity of TDOS at the Fermi level increase gradually, suggesting the progressively enhanced conductivity of W_n+1_C_n_ and W_n+1_C_n_O_2_ MXenes. Furthermore, the d-band centers of the surface W atoms for W_2_C, W_3_C_2_, and W_4_C_3_ are calculated to be −3.11, −3.73, and −3.90 eV with respect to the Fermi level, respectively, while the p-band centers of the surface O atoms for W_2_CO_2_, W_3_C_2_O_2_, and W_4_C_3_O_2_ are calculated to be −4.37, −4.57, and −4.61 eV, respectively. As the atomic layer increases, both the W d band center of W_n+1_C_n_ and the O p band center of W_n+1_C_n_O_2_ move downwards and away from the Fermi level, suggesting that the binding strength of the MXene surfaces to the Li_x_O_2_ intermediate in the Li-O_2_ batteries gradually weakens [51,52]. Therefore, increasing atomic layers of W_n+1_C_n_/W_n+1_C_n_O_2_ MXenes is not only beneficial for improving conductivity, but also weakens the adsorption of Li_x_O_2_ intermediates, thereby preventing their accumulation on the electrode surfaces.

**Figure 3:**
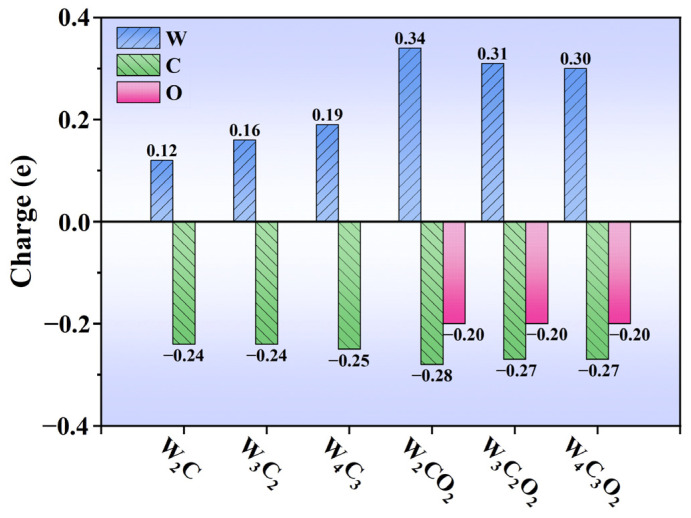
The Hirshfeld charge (in e) of surface W atoms, sublayer C atoms, and surface O groups of W_n+1_C_n_ and W_n+1_C_n_O_2_.

**Figure 4:**
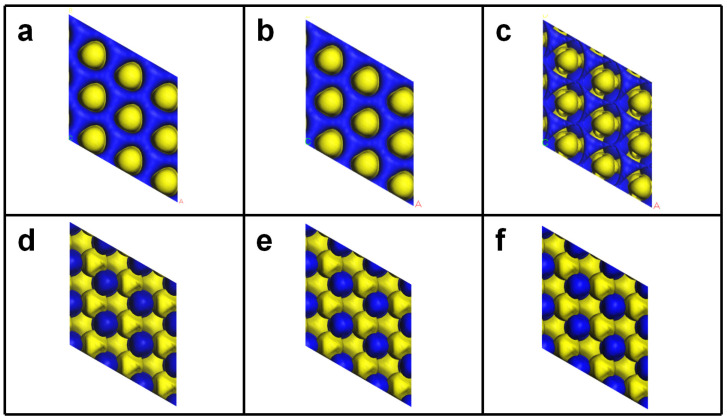
Differential electron density maps of (**a**) W_2_C, (**b**) W_3_C_2_, (**c**) W_4_C_3_, (**d**) W_2_CO_2_, (**e**) W_3_C_2_O_2_, and (**f**) W_4_C_3_O_2_.

According to Hirshfeld’s charge population analyses (Figure 3), the surface W atom of W_n+1_C_n_ MXenes carries a positive charge of 0.12–0.19 e, while the sublayer C atom has a negative charge of −0.24 e to −0.25 e, suggesting electron transfer from W to C. Therefore, the surface W atom in W_n+1_C_n_ is a positive charge center, showing electrophilicity. When the -O groups are introduced on the surface of W_n+1_C_n_, the positive charge on the W atom of W_n+1_C_n_O_2_ increases to 0.30 *e*~0.34 *e*, and the -O group has a negative charge of −0.20 e, which indicates that strong electron transfer from W to O occurs in W_n+1_C_n_O_2_. Thus, the surface O atom in W_n+1_C_n_O_2_ forms a negative charge center, which is nucleophilic. This situation can be further confirmed with the differential electron density maps. As shown in Figure 4, an electron depletion region (yellow) is observed on the surface W atoms of W_n+1_C_n_, while an electron accumulation region (blue) is located on the surface O atoms of W_n+1_C_n_O_2_, due to the different electronegativity between non-metallic oxygen and metallic W. Consequently, oxygen functionalization transforms WC MXenes from an electrophilic surface of W_n+1_C_n_ to a nucleophilic surface of W_n+1_C_n_O_2_, thereby regulating the adsorption and activation of intermediates on the MXene surfaces.

The adsorption of the Li_x_O_2_ (x = 1, 2, and 4) intermediates was examined on W_n+1_C_n_ and W_n+1_C_n_O_2_. As shown in Figure S1, the Li_x_O_2_ intermediates exhibit similar adsorption configurations on the W_n+1_C_n_ surfaces, where the surface W atom binds to the O atom in Li_x_O_2_. This is because the electrophilic W atoms on the W_n+1_C_n_ surface prefer to bind with the negatively charged O atoms in Li_x_O_2_. The strong interaction between the oxygen atom and the surface W atom promotes the activation of the O-O bond in the adsorbed Li_x_O_2_*. Consequently, the O-O bonds in the adsorbed Li_x_O_2_* on W_n+1_C_n_ are apparently longer than the Li-O bonds (see Table S3), which hinders the deintercalation of lithium during the charge–discharge processes. Alternatively, the surface O atoms of W_n+1_C_n_O_2_ directly adsorb the Li atoms of Li_x_O_2_ (see Figure S2), because the nucleophilic surface O atoms tend to bind with positively charged Li atoms. This results in a shortened O-O bond and an elongated Li-O bond after Li_x_O_2_ adsorbs on the surface of W_n+1_C_n_O_2_ (Table S4), thereby facilitating the deintercalation of lithium during the charge–discharge processes. Thus, oxygen functionalization can effectively improve the lithium deintercalation process on the WC MXene surfaces.

The adsorption energies of the Li_x_O_2_ (x = 1, 2, and 4) intermediates on W_n+1_C_n_ and W_n+1_C_n_O_2_ are listed in Table S2. Compared with the W surface of W_n+1_C_n_, the O surface of W_n+1_C_n_O_2_ exhibits weaker adsorption towards the Li_x_O_2_ intermediates. This is in good accordance with previous theoretical reports, where the oxide layer behaves as a passivation layer on the TiC(111), ZrC(111), α-MoC(001), and Mo_2_C(001) systems upon Li_2_O_2_ adsorption [52]. Furthermore, with an increasing number of atomic layers, the adsorption energy of the Li_x_O_2_ intermediates on both W_n+1_C_n_ and W_n+1_C_n_O_2_ MXenes is gradually weakened. The situation can be attributed to the downward shift of the d-band centers of surface W atoms in W_n+1_C_n_ and the p-band centers of surface O atoms in W_n+1_C_n_O_2_ with the increasing atomic layers. As shown in Figure 2, the d-band center of surface W atoms is shifted from −3.11 eV in W_2_C to −3.90 eV in W_4_C_3_, while the p-band center of surface O atoms is shifted from −4.37 eV in W_2_CO_2_ to −4.61 eV in W_4_C_3_O_2_. The downward shift of the band center increases the electron filling on the anti-bonding states between MXenes and the adsorbate, resulting in a weakened binding interaction. Hence, both oxygen functionalization and increasing atomic layers can weaken the interaction between WC MXenes and the Li_x_O_2_ intermediates, avoiding their accumulation caused by their excessive adsorption.

### 3.2. Evaluation of Catalytic Activity

Based on the reported experimental and theoretical results [2,3,47], we investigated three surface reaction steps (Equations (2)–(4)) to simulate the ORR/OER process on WC MXenes. During the discharge process, the O_2_* species on the surface of WC MXene cathodes undergoes initial metallization with (${Li}^{+}+e^{-}$) to form adsorbed LiO_2_*. Subsequently, LiO_2_* undergoes a second metallization with (${Li}^{+}+e^{-}$) to generate Li_2_O_2_*. Finally, Li_2_O_2_* further reacts with (${Li}^{+}+e^{-}$) to yield the final product (Li_2_O)_2_*. The OER at the cathode of a Li-O_2_ battery during charging is essentially the reverse process of the aforementioned ORR. In the OER process, the ultimate adsorption product (Li_2_O)_2_* gradually decomposes into O_2_*, which then dissociates from the surface of WC MXenes.

(2) $$O_{2}{\;\;}^{*}+{Li}^{+}+e^{-}↔{LiO}_{2}{\;\;}^{*}$$

(3) $${LiO}_{2}{\;\;}^{*}+{Li}^{+}+e^{-}↔{Li}_{2}O_{2}{\;\;}^{*}$$

(4) $${Li}_{2}O_{2}{\;\;}^{*}+2{Li}^{+}+2e^{-}↔{\left({Li}_{2}O\right)}_{2}{\;\;}^{*}$$

**Figure 5:**
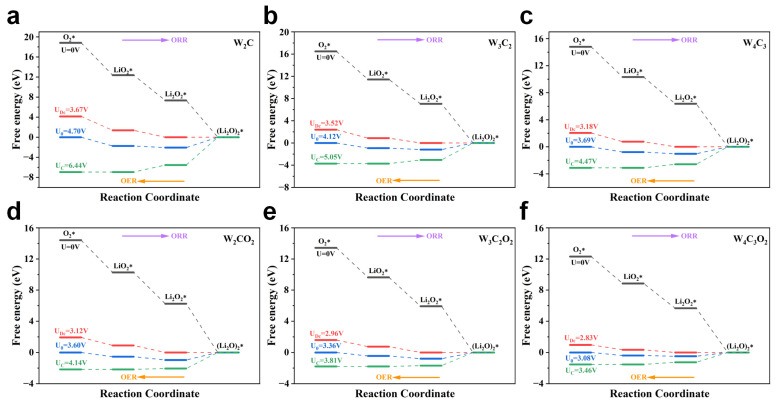
Free energy diagram of the ORR/OER process for Li_x_O_2_ intermediates on (**a**) W_2_C, (**b**) W_3_C_2_, (**c**) W_4_C_3_, (**d**) W_2_CO_2_, (**e**) W_3_C_2_O_2_, and (**f**) W_4_C_3_O_2_. * indicates that the intermediate is in an adsorbed state.

Subsequently, we constructed the free energy diagram to illustrate the ORR/OER process on W_n+1_C_n_ and W_n+1_C_n_O_2_ (Figure 5). The purple arrows in the graph represent the discharge-oriented ORR process from left to right, and the orange arrows indicate the charging-driven OER process from right to left. The nucleation of (Li_2_O)_2_ on WN MXenes follows three steps: O_2_* → LiO_2_*→ Li_2_O_2_*→ (Li_2_O)_2_*. Notably, at open circuit voltage (*U* = 0 V), both W_n+1_C_n_ and W_n+1_C_n_O_2_ (the black path in Figure 5) exhibit a downhill trend in their free energy profiles for all three metallization steps of ORR, suggesting the spontaneous nucleation of (Li_2_O)_2_ on the surface of WC MXenes. Conversely, during the reverse OER process, the decomposition of (Li_2_O)_2_ is endothermic on both W_n+1_C_n_ and W_n+1_C_n_O_2_ MXenes. Furthermore, the whole free energy change (ΔG(O_2_*→(Li_2_O)_2_*)) of the O_2_*→(Li_2_O)_2_* process decreases with the increasing atomic layers of W_n+1_C_n_ and W_n+1_C_n_O_2_. This is because the WC MXenes exhibit a weakened affinity towards Li_x_O_2_ intermediates as the number of atomic layers increases. These findings suggest that as the number of atomic layers increases, it becomes easier for (Li_2_O)_2_ products to undergo decomposition on the WC MXene surfaces. Moreover, compared to the pristine W_n+1_C_n_, ΔG(O_2_→(Li_2_O)_2_*) for the oxygen-functionalized W_n+1_C_n_O_2_ is significantly reduced, which is further beneficial for the (Li_2_O)_2_ decomposition. Therefore, both increasing atomic layers and oxygen functionalization can promote the de-lithiation of (Li_2_O)_2_ products, thereby accelerating the electrochemical process during charging.

In Figure 5, *U*_Dc_ represents the maximum discharge potential driving the energy of all ORR steps to exhibit a downward trend along the red path from left to right, while *U_C_* represents the minimum charging potential driving the energy of all OER steps to decrease along the green path from right to left. The equilibrium potential *U*_0_ applied in the blue path facilitates achieving equilibrium in the electrochemical ORR/OER process. With an increase in atomic layer number, the ΔG(O_2_*→(Li_2_O)_2_*) of W_n+1_C_n_ MXenes gradually decreases, and therefore the required *U_0_* decreases as well. The values of *U_0_* are calculated to be 4.70, 4.12, and 3.70 V for W_2_C, W_3_C_2_, and W_4_C_3_, respectively (Figure 5a–c). After surface oxygen functionalization, the ΔG(O_2_*→(Li_2_O)_2_*) of W_n+1_C_n_O_2_ further decreases significantly, and thus the corresponding *U*_0_ for W_2_CO_2_, W_3_C_2_O_2_, and W_4_C_3_O_2_ is decreased to be 3.60, 3.36, and 3.08 V, respectively (Figure 5d–f). Consequently, the W_4_C_3_O_2_ MXene has a minimum *U*_0_ among W_n+1_C_n_ and W_n+1_C_n_O_2_ MXenes.

When *U*_0_ is applied to the electrochemical processes on W_n+1_C_n_ and W_n+1_C_n_O_2_, the formation steps of Li_2_O_2_* and LiO_2_* along the ORR pathway are still downhill in the free energy profiles (the blue path in Figure 5). However, the last (Li_2_O)_2_* formation step shows an upward trend, suggesting it forms the rate-determining step (RDS) of the ORR pathway on both W_n+1_C_n_ and W_n+1_C_n_O_2_. Compared to the downhill step of the (Li_2_O)_2_* decomposition, the decomposition of Li_2_O_2_* and LiO_2_* is uphill along the OER pathway in the free energy profiles. Furthermore, the decomposition of LiO_2_* requires a higher energy input than that of Li_2_O_2_*, suggesting that it serves as the RDS of OER on W_n+1_C_n_ and W_n+1_C_n_O_2_.

**Figure 6:**
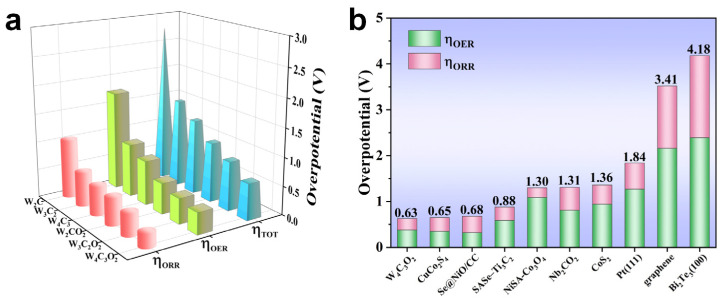
(**a**) The ORR (*η*_ORR_), OER (*η*_OER_), and total (*η*_TOT_) overpotentials for WC MXenes. (**b**) Comparison of overpotentials for W_4_C_3_O_2_ MXene with other materials.

The overpotential η is defined as the minimum $\left|U-U_{0}\right|$ that makes all the electrochemical steps downhill in free energy and serves as a crucial indicator for assessing the catalytic performance of a catalyst [51]. The smaller the value of η, the lower the actual voltage required to achieve a target current density, resulting in reduced energy consumption and enhanced catalytic activity [45,53]. In this study, we calculated the ORR ($\eta_{ORR}$), OER ($\eta_{OER}$), and total ($\eta_{TOT}$) overpotentials by $\eta_{ORR}=U_{0}-U_{Dc}$, $\eta_{OER}=U_{C}-U_{0}$, and $\eta_{TOT}=\eta_{ORR}+\eta_{OER}$, respectively. Detailed data on overpotentials are presented in Figure 6. The overpotentials ${{(\eta }_{OER}/\eta }_{ORR}/\eta_{TOT})$ of the pristine W_n+1_C_n_ follow the order: W_4_C_3_ (0.78 V/0.51 V/1.29 V) < W_3_C_2_ (0.93 V/0.60 V/1.53 V) < W_2_C (1.74 V/1.03 V/2.77 V), suggesting that the W_n+1_C_n_ MXenes show a decrease trend in overpotentials with an increasing number of atomic layers. After the WC surfaces are covered with O groups, there is a significant decrease in overpotentials. The values of $\eta_{OER}$, $\eta_{ORR}$, and $\eta_{TOT}$ are decreased in the order of W_4_C_3_O_2_ (0.38 V/0.25 V/0.63 V) < W_3_C_2_O_2_ (0.45 V/0.39 V/0.84 V) < W_2_CO_2_ (0.54 V/0.48 V/1.02 V). Moreover, the values of $\eta_{OER}$ for W_n+1_C_n_ and W_n+1_C_n_O_2_ MXenes are higher than those of $\eta_{ORR}$, indicating the slower kinetics of the OER during the charging process, which may lead to poor cyclic stability [54]. This is attributed to the strong adsorption of the Li_x_O_2_ produced during the discharge process, which makes it difficult to reversibly decompose, resulting in continuous accumulation [55,56]. Among all considered WC MXenes, W_4_C_3_O_2_ exhibits the lowest OER, ORR, and total overpotentials (0.38, 0.25, and 0.63 V). Furthermore, it is worth noting that the overpotentials ($\eta_{OER}$, $\eta_{ORR}$, and $\eta_{TOT}$) of W_4_C_3_O_2_ are lower than those of Nb_2_CO_2_ MXene (0.81, 0.50, and 1.31 V) [57], Se@NiO/CC (0.32, 0.36, and 0.68 V) [58], SASe–Ti_3_C_2_ (0.59, 0.29, and 0.88 V) [59], CuCo_2_S_4_ (0.35, 0.30, and 0.65 V) [60], NiSA-Co_3_O_4_ (1.09, 0.21, and 1.30 V) [12], CoS_2_ (0.89, 0.47, and 1.36 V) [61], and other recently reported two-dimensional materials [45,62,63]. These suggest that the W_4_C_3_O_2_ MXene shows excellent catalytic performance as a cathode catalyst for Li-O_2_ batteries.

**Figure 7:**
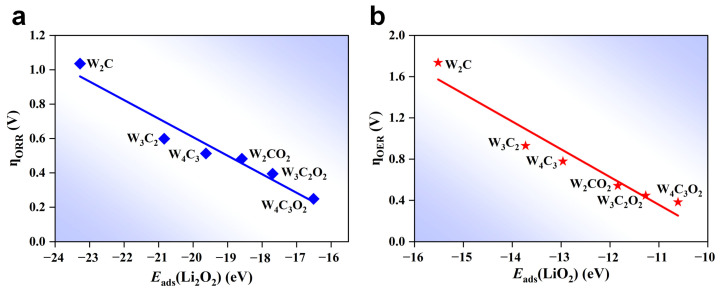
(**a**) The ORR overpotential (*ƞ_ORR_*) as the function of the adsorption energy of Li_2_O_2_ (*E*_ads_(Li_2_O_2_)). (**b**) The OER overpotential (*ƞ*_OER_) as the function of the adsorption energy of LiO_2_ (*E*_ads_(LiO_2_)).

To further investigate the relationship between the adsorption property of Li_x_O_2_ and the overpotentials, we plotted the correlation between the adsorption energy (*E*_ads_) of RDS intermediates and $\eta_{ORR}$*/*$\eta_{OER}$ in the ORR/OER process for WC MXenes (Figure 7). In the ORR process, the reduction of Li_2_O_2_* to (Li_2_O)_2_* serves as the RDS on both W_n+1_C_n_ and W_n+1_C_n_O_2_ MXenes, and thus the adsorption energy of Li_2_O_2_* (*E*_ads_(Li_2_O_2_*)) plays a key role in $\eta_{ORR}$. As depicted in Figure 7a, there is a linear correlation between *E*_ads_(Li_2_O_2_*) and $\eta_{ORR}$ for WC MXenes. The values of $\eta_{ORR}$ decrease with the decrease of the adsorption energy of Li_2_O_2_* on W_n+1_C_n_ and W_n+1_C_n_O_2_ MXenes. Reducing the adsorption strength of Li_2_O_2_* is beneficial for further metallization to produce (Li_2_O)_2_*, resulting in a reduced value of the corresponding $\eta_{ORR}$ for WC MXenes. In the OER process, the decomposition of LiO_2_* into $O_{2}{\;\;}^{*}$ acts as the RDS on WC MXenes. Thus, the adsorption energy of LiO_2_^*^ (*E*_ads_(LiO_2_*)) is important in determining $\eta_{OER}$. As shown in Figure 7b, *E*_ads_(LiO_2_*) and $\eta_{OER}$ exhibit a linear relationship for W_n+1_C_n_ and W_n+1_C_n_O_2_ MXenes. A weaker *E*_ads_(LiO_2_*) can promote the decomposition of LiO_2_*, leading to a lower value of the corresponding $\eta_{OER}$. These findings demonstrate that the reduced adsorption energy of the RDS intermediates (LiO_2_* and Li_2_O_2_*) has a positive effect on reducing overpotentials. By lowering the energy band center, oxygen functionalization and increasing the atomic layer can effectively reduce the adsorption strength of the RDS intermediates (Li_2_O_2_* and LiO_2_*), thereby reducing the ORR and OER overpotentials.

## 4. Conclusions

In this work, the models of pristine W_n+1_C_n_ and oxygen-functionalized W_n+1_C_n_O_2_ MXenes were constructed, and their catalytic performance as cathodes for Li-O_2_ batteries was evaluated using first principles calculations. The TDOS analyses at the Fermi level confirm the excellent electrical conductivity of both W_n+1_C_n_ and W_n+1_C_n_O_2_, which is further enhanced with increasing atomic layers. The oxygen functionalization alters the electronic properties of WC MXenes from the electrophilic W surface of W_n+1_C_n_ to the nucleophilic O surface of W_n+1_C_n_O_2_, which facilitates the Li-O bond activation and thus promotes Li deintercalation during the charge–discharge process. Compared to the pristine W_n+1_C_n_, the oxygen-functionalized W_n+1_C_n_O_2_ has a significantly reduced adsorption energy towards Li_x_O_2_, resulting in lower overpotentials of $\eta_{OER},\eta_{ORR},and\;\eta_{TOT}$. As the number of atomic layers in WC MXenes increases, the adsorption energy of Li_x_O_2_ is further decreased, leading to a reduction in $\eta_{OER}$, $\eta_{ORR}$, and $\eta_{TOT}$. The O-terminated W_4_C_3_O_2_ MXene shows superior electrical conductivity and remarkably low overpotentials (0.38 V for $\eta_{OER}$, 0.25 V for $\eta_{ORR}$, and 0.63 V for $\eta_{TOT}$), highlighting its huge potential as a cathode catalyst for Li-O_2_ batteries. The study indicates that the WC MXenes can serve as cathode materials for Li-O_2_ batteries, and W_4_C_3_O_2_ is identified as a high-performance cathode catalyst material. This finding is of great importance for the design and manufacture of cathode catalysts used in Li-O_2_ batteries.

## Data Availability

Data will be made available on request.

## Supplementary Materials

The following supporting information can be downloaded at: https://www.mdpi.com/article/10.3390/nano14080666/s1, Figure S1: the top and side views of the adsorption configuration of LiO_2_ on WC MXenes; Figure S2: the top and side views of the adsorption configuration of Li_2_O_2_ on WC MXenes; Table S1: formation energy of W_2_C, W_3_C_2_, and W_4_C_3_; Table S2: the adsorption energy of Li_x_O_2_ on W_n+1_C_n_ and W_n+1_C_n_O_2_; Table S3: the average length of the Li-O and O-O bonds in LiO_2_/Li_2_O_2_ and the adsorbed distance of LiO_2_/Li_2_O_2_ to W_n+1_C_n_; Table S4: the average length of the Li-O and O-O bonds in LiO_2_/Li_2_O_2_ and the adsorbed distance of LiO_2_/Li_2_O_2_ to W_n+1_C_n_O_2_; Table S5: *U*_Dc_, *U*_0_, *U_C_*, $\eta_{ORR}$, $\eta_{OER}$, and $\eta_{TOT}$ for W_n+1_C_n_ and W_n+1_C_n_O_2_.
