# Supplementary material for: First Principles Study of the Structure–Performance Relation of Pristine Wn+1Cn and Oxygen-Functionalized Wn+1CnO2 MXenes as Cathode Catalysts for Li-O2 Batteries
Source: Nanomaterials (Basel). 2024 Apr 11;14(8):666. doi: 10.3390/nano14080666 (PMC11054248; doi:10.3390/nano14080666)
Supplement: Supplementary file 1 [file nanomaterials-14-00666-s001.zip › nanomaterials-2945081-supplementary.pdf]

Supporting Information

First-Principles Study of the Structure-Performance Relation of Pristine $W_{n+1}C_n$ and Oxygen-Functionalized $W_{n+1}C_nO_2$ MXenes as Cathode Catalysts for Li-O_2 Batteries

Liwei Zhu,Jiajun Wang, Jie Liu, Ruxin Wang, Meixin Lin, Tao Wang, Yuchao Zhen, Jing Xu*, Lianming Zhao*

School of Materials Science and Engineering, China University of Petroleum (East China), Qingdao, Shandong 266580, PR China

* Corresponding authors.

E-mail addresses: lmzhao@upc.edu.cn (L. Zhao), xujing@upc.edu.cn (J. Xu).

Table of contents

1. The adsorption configuration of LiO_2 on $W_{n+1}C_n$ and $W_{n+1}C_nO_2$ MXenes ···········S-3

2. The adsorption configuration of Li_2O_2 on $W_{n+1}C_n$ and $W_{n+1}C_nO_2$ MXenes··········S-4

3. Formation energy of W_2C, W_3C_2, and W_4C_3.············· ···S-5

4. The adsorption energy of Li_xO_2 on $W_{n+1}C_n$ and $W_{n+1}C_nO_2$ ···································S-6

5. The average length of the Li−O and O−O bonds in LiO_2/Li_2O_2 and the adsorbed distance of LiO_2/Li_2O_2 on $W_{n+1}C_n$···S-7

6. The average length of the Li−O and O−O bonds in LiO_2/Li_2O_2 and the adsorbed distance of LiO_2/Li_2O_2 on $W_{n+1}C_nO_2$ ··S-8

7. U_{Dc}, U_0, U_C, η_{ORR}, η_{OER}, and η_{TOT} for $W_{n+1}C_n$ and $W_{n+1}C_nO_2$.······· ··················S-9

Fig. S1. The top and side views of the adsorption configuration of LiO_2 on (a1) W_2C, (a2) W_3C_2, (a3) W_4C_3, (b1) W_2CO_2, (b2) $W_3C_2O_2$, and (b3) $W_4C_3O_2$ MXenes.

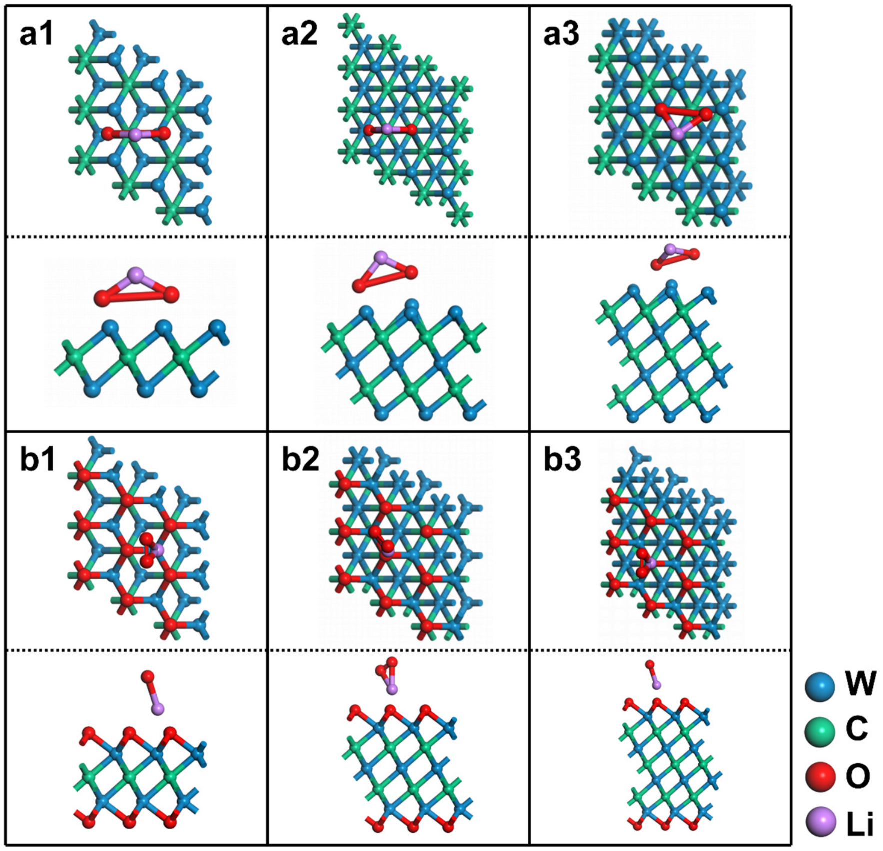

Fig. S2. The top and side views of the adsorption configuration of Li_2O_2 on (a1) W_2C, (a2) W_3C_2, (a3) W_4C_3, (b1) W_2CO_2, (b2) $W_3C_2O_2$, and (b3) $W_4C_3O_2$ MXenes.

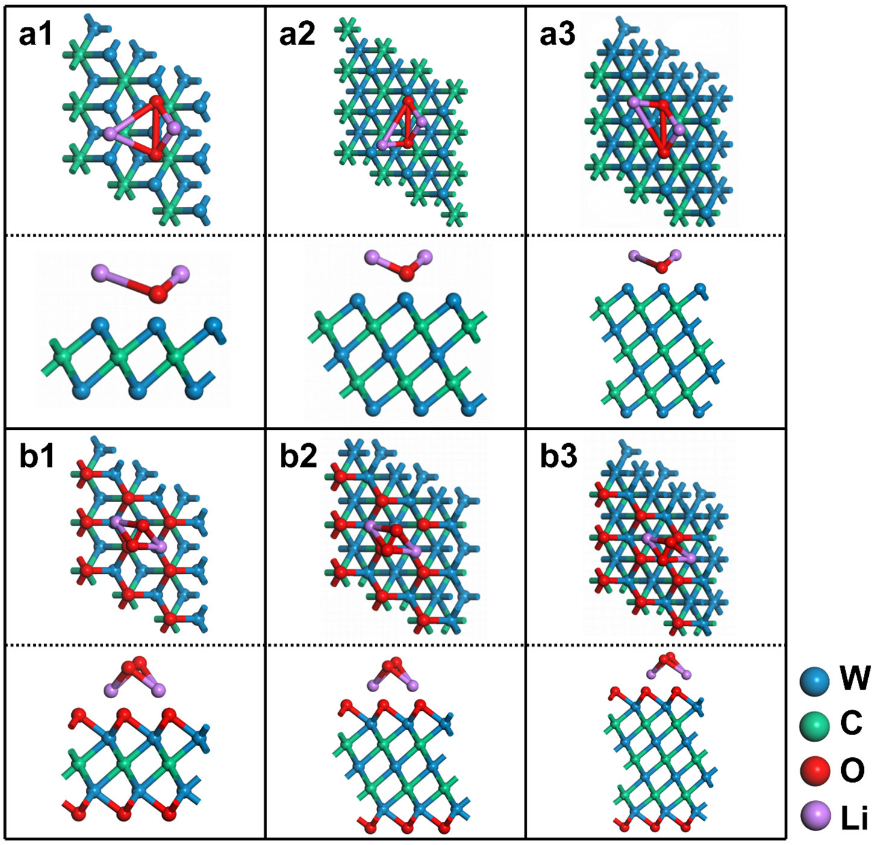

Table S1. Formation energy E_f (in eV) of W_2C, W_3C_2, and W_4C_3.

Materials	E_f/eV
W_2C	−3.48
W_3C_2	−3.35
W_4C_3	−3.33

Table S2. The adsorption energy E_{ads} (in eV) of Li_xO_2 (x = 1, 2, and 4) on $W_{n+1}C_n$ and $W_{n+1}C_nO_2$.

Materials	$E_{ads}(O_2)$	$E_{ads}(LiO_2)$	$E_{ads}(Li_2O_2)$	$E_{ads}((Li_2O)_2)$
W_2C	−6.36	−15.52	−23.28	−36.05
W_3C_2	−5.98	−13.73	−20.84	−33.33
W_4C_3	−5.77	−12.96	−19.63	−31.44
W_2CO_2	−4.98	−11.84	−18.59	−30.27
$W_3C_2O_2$	−4.74	−11.27	−17.70	−29.06
$W_4C_3O_2$	−4.43	−10.61	−16.51	−27.62

Table S3. The average length (in Å) of the Li−O (d_{Li-O}) and O−O bonds (d_{O-O}) in LiO_2/Li_2O_2 and the adsorbed distance of LiO_2/Li_2O_2 on $W_{n+1}C_n$ (d_{Li-sub}/d_{O-sub}).

	LiO_2				Li_2O_2			
Distance	Free	W_2C	W_3C_2	W_4C_3	Free	W_2C	W_3C_2	W_4C_3
d_{O-O}	1.372	3.153	3.233	2.703	1.597	2.849	2.876	2.911
d_{Li-O}	1.774	1.845	2.006	1.946	1.718	2.491	2.344	2.396
d_{Li-sub}	\	2.512	2.582	2.461	\	2.244	2.204	2.220
d_{O-sub}	\	1.628	1.416	1.581	\	1.357	1.338	1.409

Table S4. The average length (in Å) of the Li−O (d_{Li-O}) and O−O bonds (d_{O-O}) in LiO_2/Li_2O_2 and the adsorbed distance of LiO_2/Li_2O_2 on $W_{n+1}C_nO_2$ (d_{Li-sub}/d_{O-sub}).

	LiO_2				Li_2O_2			
Distance	Free	W_2CO_2	$W_3C_2O_2$	$W_4C_3O_2$	Free	W_2CO_2	$W_3C_2O_2$	$W_4C_3O_2$
d_{O-O}	1.372	1.274	1.286	1.304	1.597	1.336	1.312	1.303
d_{Li-O}	1.774	2.126	2.053	1.977	1.718	2.112	2.172	2.203
d_{Li-sub}	\	1.435	1.446	1.499	\	1.322	1.262	1.284
d_{O-sub}	\	3.373	3.246	3.322	\	2.672	2.674	2.722

Table S5. U_{Dc}, U_0, U_C, η_{ORR}, η_{OER}, and η_{TOT} (in V) for $W_{n+1}C_n$ and $W_{n+1}C_nO_2$.

Materials	U_{Dc}	U_0	U_c	η_{ORR}	η_{OER}	η_{TOT}
W_2C	3.67	4.70	6.44	1.04	1.74	2.77
W_3C_2	3.52	4.12	5.05	0.60	0.93	1.53
W_4C_3	3.18	3.69	4.47	0.51	0.78	1.29
W_2CO_2,	3.12	3.60	4.14	0.48	0.54	1.02
$W_3C_2O_2$	2.96	3.36	3.81	0.39	0.45	0.84
$W_4C_3O_2$	2.83	3.08	3.46	0.25	0.38	0.63
